# Improving shared decision‑making between paediatric haematologists, children with sickle cell disease, and their parents: an observational post-intervention study

**DOI:** 10.1007/s00431-025-06241-2

**Published:** 2025-06-12

**Authors:** Ricardo O. Wijngaarde, Samantha C. Gouw, Dirk T. Ubbink

**Affiliations:** 1 Emma Children’s Hospital, Amsterdam University Medical Center, AMC, Meibergdreef 9, 1105 AZ Amsterdam, The Netherlands; 2 Department of Surgery, Amsterdam University Medical Center, University of Amsterdam, Meibergdreef 9, 1105 AZ Amsterdam, The Netherlands; 3 Department of Pediatric Hematology, Amsterdam University Medical Center, University of Amsterdam, Meibergdreef 9, 1105 AZ Amsterdam, The Netherlands

**Keywords:** Shared decision-making, Paediatrics, Sickle cell disease, Triadic, Chronic disease, OPTION-instrument, SDM-Q-9 questionnaire, SDM-Q-Doc questionnaire

## Abstract

Children with sickle cell disease (SCD) suffer from a chronic disease that can lead to serious co-morbidity and impacts their quality of life. During the course of their disease, a variety of health-related decisions need to be made for and by SCD-patients, depending on their age and health status, together with their parents and paediatric haematology clinicians. Shared decision-making (SDM) may improve health outcomes of chronically ill children but is still not commonly applied. We assessed the level of SDM among paediatric haematologists after the introduction of SDM interventions. An observational post-intervention study was conducted in a paediatric outpatient clinic of a university hospital. After an SDM consultation training of the three paediatric haematologists and introduction of SDM-supporting tools for both paediatricians and (parents of) patients with SCD, two evaluators independently and objectively analysed the level of patient involvement in decision-making from audio-recordings of the consultations using the OPTION-5 instrument. SDM-Q-9 and SDM-Q-Doc questionnaires were used to measure the level of SDM as perceived by patients/parents and paediatricians, respectively. Scores were expressed as a percentage, ranging from 0% (no SDM observed) to 100% (exemplary level of SDM). Participants were 9 female and 9 male patients between 4 months and 17 years old, with a median age of 7.5 years (Interquartile Range [IQR] 2.5–12). Eighteen consultations (six per paediatrician) in which a decision was to be made about SCD treatment options were analysed. Median OPTION-5 score was 50 (IQR 40–65%). Median SDM-Q-9 and SDM-Q-Doc scores were 73% (IQR 52.2–91) and 62.2% (IQR 55.6–71.1), respectively.

*Conclusion:* After the introduction of SDM training and tools, paediatric haematologists reached a moderately good level of SDM. This level had doubled as compared to the baseline level, as assessed in a previous study.

**Table:** 

**What is Known:** • Children who suffer from sickle cell disease (SCD) are vulnerable to health inequities and suboptimal health outcomes. Hence, SDM seems an appropriate method of care for these children. • SDM tools and training may help paediatricians and children participate in a collaborative decision-making process about the children’s preferred treatment options and improve their health outcomes.
**What is New:** • After SDM training and decision support aids for paediatricians and patients, the level of involvement in the decision-making process by (the parents of) patients suffering from SCD reached a moderately good level. • A difference persists between paediatricians’ perceived level of involving the child and parents in a shared decision-making process and the observed level of involvement.

**What is Known:**

• Children who suffer from sickle cell disease (SCD) are vulnerable to health inequities and suboptimal health outcomes. Hence, SDM seems an appropriate method of care for these children.

• SDM tools and training may help paediatricians and children participate in a collaborative decision-making process about the children’s preferred treatment options and improve their health outcomes.

**What is New:**

• After SDM training and decision support aids for paediatricians and patients, the level of involvement in the decision-making process by (the parents of) patients suffering from SCD reached a moderately good level.

• A difference persists between paediatricians’ perceived level of involving the child and parents in a shared decision-making process and the observed level of involvement.

## Introduction

The three main principles of the United Nations Convention on the Rights of the Child (UNCRC) are *Protection*, *Provision*, and *Participation* [1, 2]. The UNCRC principal rule is embodied by its article 3, which exemplifies the child’s best interest as the guiding principle for all decisions that affect a child’s well-being. Along with the Right to health, the Right to be heard, and the Right to participate, as stated by the UNCRC, this interdependent set of principles constitutes a moral and legal foundation for a default application of shared decision making (SDM) in paediatric care [3].

Studies have shown that SDM in paediatrics leads to better health outcomes [4, 5]. All the more for chronically ill children, SDM seems the appropriate decision-making strategy as many health- and care-related decisions have to be made during the course of their disease, while the child’s decision-making capacity and maturity evolve, as do their healthcare needs and preferences [6–9]. This evolution changes the level of involvement of all stakeholders involved from a dyadic (parent/caregiver–paediatrician) to a triadic (patient-parent–paediatrician) communication style and back to a dyadic (patient–paediatrician) collaborative and SDM process [6].

To live up to the healthcare professionals’ standard of Good Clinical Practice, the quality and consistency of the information exchange in the patient-parent-paediatrician encounter needs to be assessed [10]. Also, influencing factors need to be taken into account, like the parents’ and patients’ (real and perceived) level of health literacy, language- and or cultural barriers, the importance of risk-communication, as well as paediatricians’ awareness of (the risk of) bias amongst all stakeholders in this realm [11, 12, 12–15].

In a previous study in 2021–2022 on SDM among paediatric haematologists and children between 2 and 17 years of age and suffering from sickle cell disease (SCD), the baseline level of SDM was assessed and showed room for improvement [16]. Our present goal was to explore if and to what extent SDM interventions could affect the level of SDM during clinical encounters. Hence, the level of SDM was investigated again in SCD patients at the same department of paediatric haematology, after having received individual and group SDM training and personalized feedback cards before consultation, as well as child-friendly decision-making support tools to be used during consultation.

## Methods

This observational post-intervention study was executed to assess the level of SDM at the Paediatric Haematology outpatient department of the Amsterdam University Medical Center (UMC), being an expert centre for SCD patients. This study was reported along the STROBE [17].

### Participants

#### Patients

(Parents of) patients suffering from SCD and visiting the paediatric outpatient clinic of the Amsterdam UMC were eligible for participation. Inclusion criteria were as follows: children younger than 18 years, suffering from SCD, and facing a health-related decision about pneumococcal vaccines, dietary changes and/or hydroxyurea treatment decisions. All participants signed informed consent.

#### Paediatricians

The full team of three haematology paediatricians, who also participated in the baseline study, were again invited for this study.

### Intervention

The multifaceted intervention in this study consisted of group and individual SDM trainings and introducing SDM tools, as applied in earlier studies [18]. The training phase was conducted in two separate stages.

In the first stage (March 2023), the team of haematologic specialists, the head paediatric haematology nurse, and a paediatric nurse-specialist from the SCD outpatient clinic took part in a half-day group SDM communication training. The training was led by a medical psychologist and included simulated patient consultations with a professional actor, based on cases from their own experience.

Stage 2 of the SDM training (May–June 2023) consisted of group feedback as well as individual SDM training based on the analysis of previous audio-recordings of their own consultations. These individual feedback sessions took between 45 and 60 min.

**Fig. 1:**
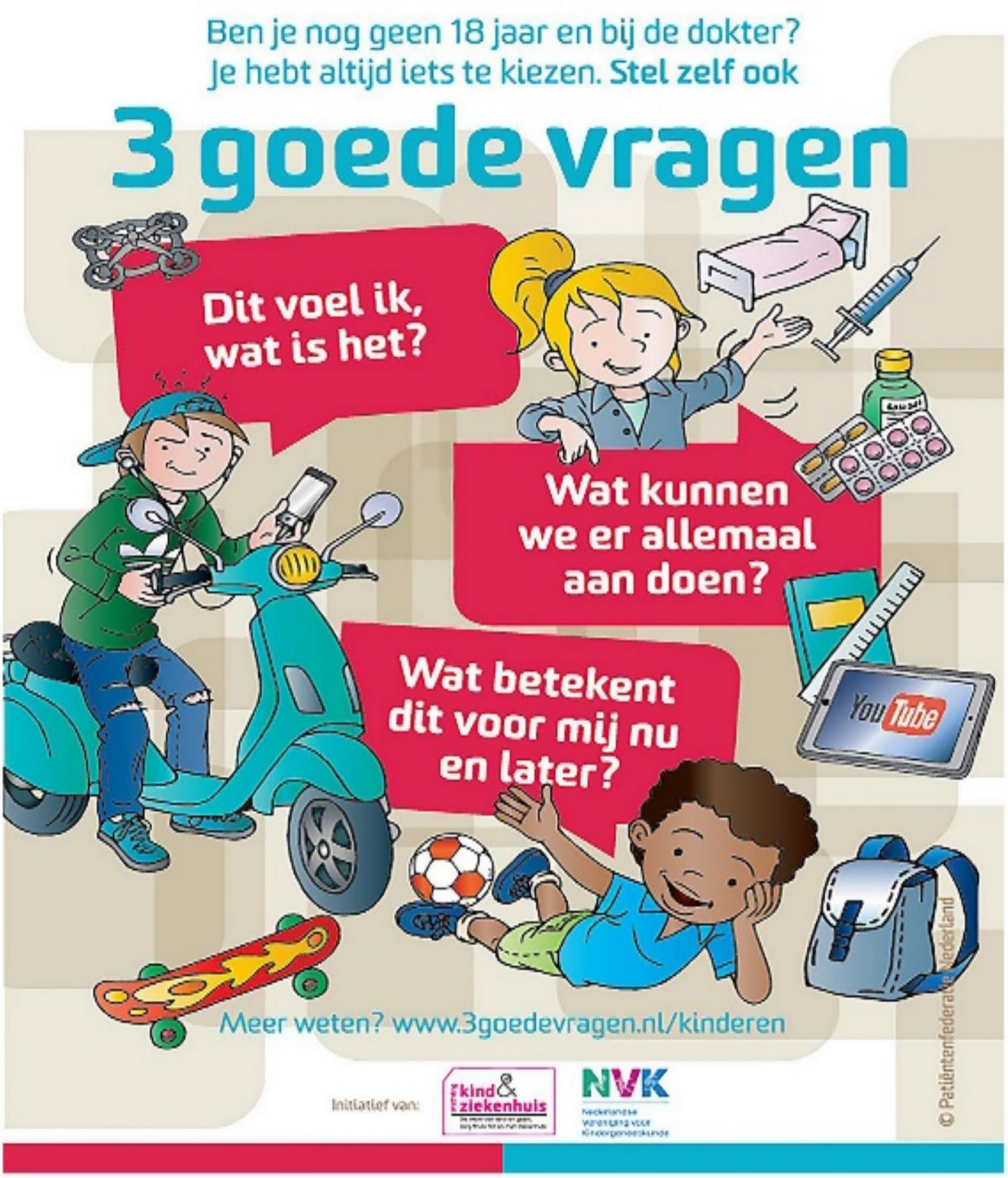
3 Goede Vragen voor Kinderen (3 Good Questions for Children), which can be translated as (1) This is what I feel, what is it?, (2) What can we do about it?, (3) How will this affect me now and later? Copyright: Patiëntenfederatie Nederland. St. Kind & Ziekenhuis, Nederlandse Vereniging voor Kindergeneeskunde

**Fig. 2:**
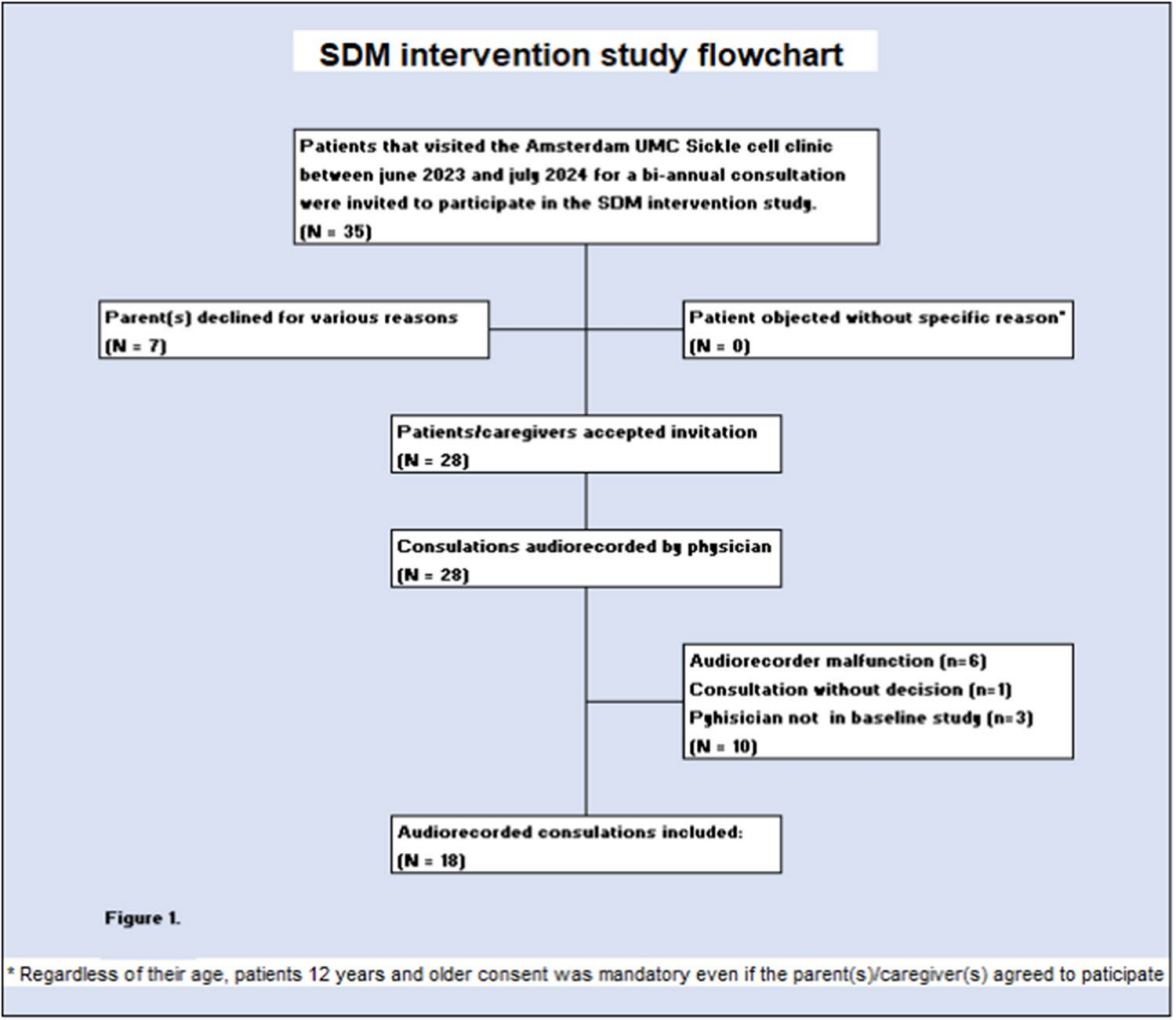
Flowchart of consultation inclusion

SDM tools comprised personalized SDM cards for each paediatric haematologist to be used during their consultations. Each card highlighted the top-3 of areas in which the most improvement could be made in the patient/caregiver encounter. Also, the ‘*3 Good Questions for Children*’ cards [19], derived from a similar card for adult patients [20, 21], were introduced during paediatric haematologists’ staff meetings preceding the SDM training (Fig. 1). These cards contain three questions for children to contemplate shortly before visiting their paediatrician. This allowed all stakeholders to initiate and guide a decision-making conversation about treatment options (Fig. 1). Physicians decided who would be a potential participant, based on whether or not a health-related decision was expected to be made during the consultation and informed the researcher in the week preceding or on the same day of the consultation (Fig. 2).

### SDM questionnaires and measures

To measure the level of a child’s and/or parents’ involvement in the clinical decision-making process, the Observing Patient Involvement (OPTION-5) instrument was used to objectively score audio-recordings of the clinical consultations [22]. This instrument has shown to have strong psychometric properties and to be less burdensome than the original OPTION-12 instrument from which it had been derived [23, 25]. The OPTION-5 instrument addresses five essential elements of the SDM process, i.e., awareness that a decision needs to be made (item 1), assurance that the patient will be well-informed to decide together (item 2), explanation of treatment options, including risks and benefits (item 3), elicitation of patients’ preferences (item 4), and deciding together about the most appropriate treatment option (item 5) [22]. Item scores range from 0 (no SDM observed) to 4 (exemplary SDM effort). The total OPTION-5 score ranges from a minimum of 0 to a maximum of 20 points.

As opposed to the observed extent of patient involvement with the OPTION-5 measurement, the subjectively perceived levels of SDM by the child/caregiver and paediatrician, were measured with the validated Dutch SDM Q-9 and SDM Q-Doc questionnaires [26], respectively (Table 1). These scores range from a minimum of 0 to a maximum of 45.

**Table 1:**
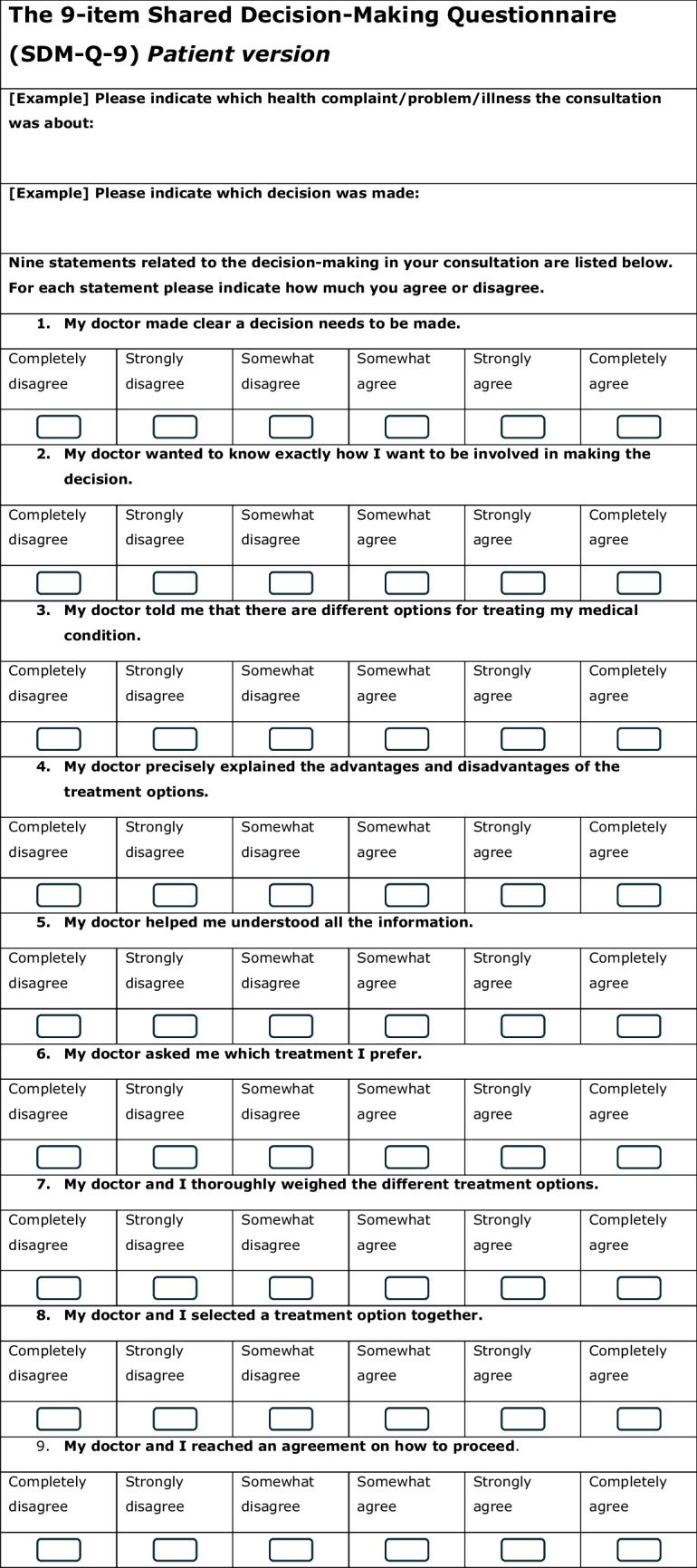

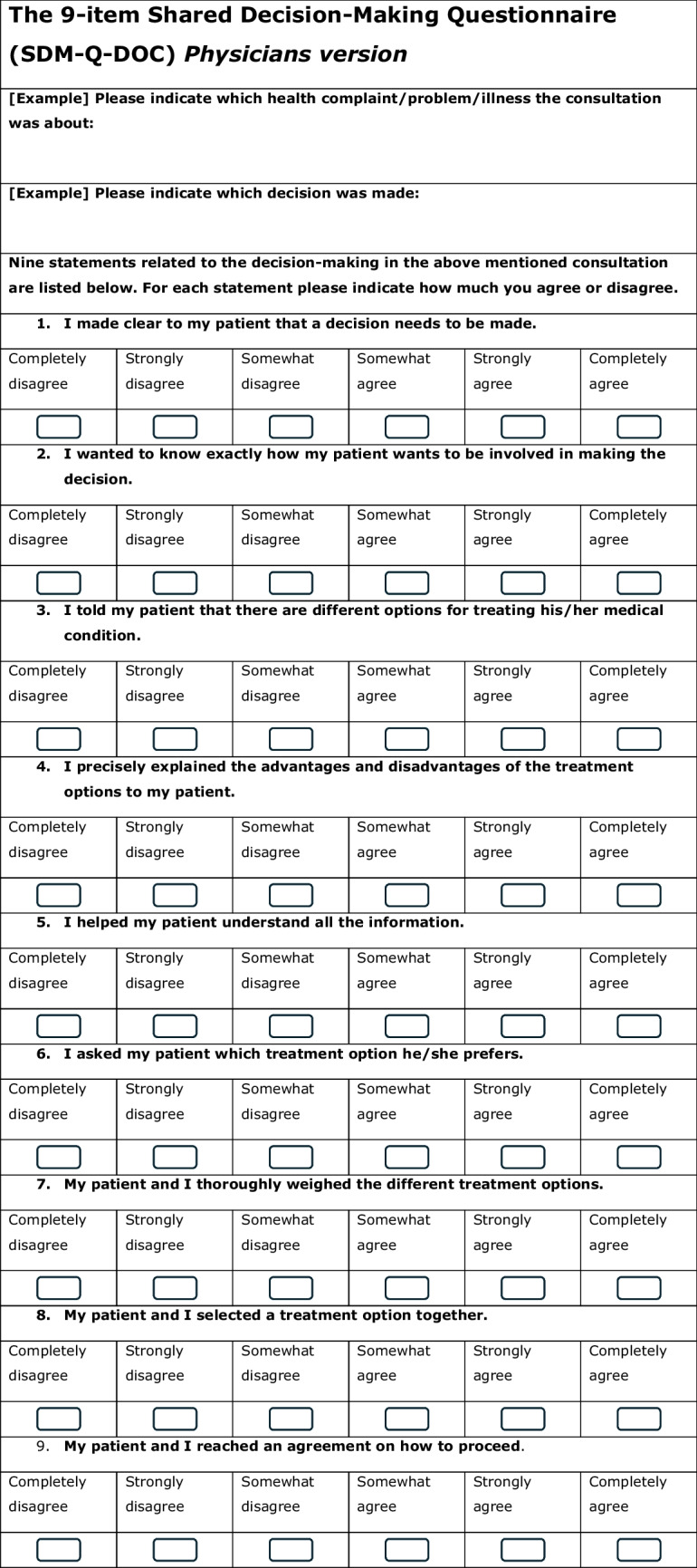
SDM-Q-9 and SDM-Q-Doc questionnaires

### Study conduct

From August 2023 to July 2024, eligible children with SCD and their parents visiting the paediatric outpatient clinic were invited to consent to participate. After obtaining informed consent, the participants’ demographic data, including age and gender, were collected. Also, a research assistant introduced and explained the ‘*3 Good Questions for Children*’ decision-making support tool and explained to the patient and parents and was used consistently during consultation.

Consultations between paediatrician and child, parent and/or caregiver, were audio-recorded with a handheld voice-recorder (Philips Voicetracer DVT 1250). Six consultations per paediatrician were recorded to obtain sufficiently reliable individual SDM scores [27]. Immediately after consultation, the children or their parents—if children were below the age of twelve years—were asked to fill in the SDM-Q-9 questionnaire and paediatricians were asked to complete the SDM-Q-Doc on paper.

### Data analysis

OPTION-5, SDM-Q-9, and SDM Q-Doc scores were expressed as percentages of the maximum score, ranging from 0% (no SDM observed) to 100% (exemplary SDM observed). Descriptive statistical analysis comprised calculating mean questionnaire scores with 95% confidence intervals (95%CI) or medians and interquartile ranges (IQR) in case of non-normal distribution.

For objective and accurate determination of the OPTION-5 scores, two evaluators (RW and DU) separately rated a first series of six audiotaped consultations, after which the separate scores were compared using the OPTION-guidelines and measurement interpretation handbook [28]. By calculating the kappa value of the OPTION-5 scores for each item separately we assessed the level of inter-observer agreement. This was deemed acceptable if the lower limit of the 95%CI of the kappa value exceeded 0.6, equalling substantial agreement. Then, the remaining recordings were analysed by a single evaluator (RW).

To assess the level of agreement between the SDM-Q-9 and SDM-Q-Doc questionnaires and to determine whether the differences were systematic and constant over the full range of scores, a Bland–Altman plot was produced. Statistical analyses were performed using SPSS (IBM SPSS v. 28, Armonk, NY, USA) [29].

## Results

All three paediatric haematologists completed the training. This team had 10 to 16 years of experience as paediatricians and 6 to 33 years as paediatric haematologists (Table 2). Our patient population consisted entirely of children from first-, second-, or third-generation migrant Dutch families, from areas like Surinam, Curacao, and a range of different nationalities from the South Asian, Middle Eastern, and sub-Sahara African region. They had a median age of 7.5 years (range 4 months to 17 years) (IQR 2.5–12) with an equal number (*n* = 9) of female and male patients. Median duration of the consultations was 25:12 mm:ss (IQR 18:05–32:51), ranging from 08:22 to 45:27 mm:ss (Table 2). Two consultations were conducted digitally (Table 2).

**Table 2:** Participant and recording data

Patients		Gender			Age			
	***N***	Female		Male	Min	Max	Median	IQR
	18	9		9	4 months	17 years	7.5 years	2.5–12 years
**Paediatricians**		**Gender**			**Experience as paediatrician (yrs)**			
	***N***	Female			Pediatr. 1	Pediatr. 2	Pediatr. 3	
	3	3			33	12	10	
**Consultation**					**Experience as paediatric haematologist (yrs)**			
With parent/caregivers			Patient alone		Pediatr. 1	Pediatr.2	Pediatr.3	
	***N***							
	17		1		28	9	6	
**Audio-recordings**			**Location**		**Duration (minutes:seconds)**			
	***N***	In clinic	Teleconsult		Min	Max	Median	IQR
	18	16	2		08:22	45:27:00	25:12:00	18:05–32:51 s

### OPTION-5

**Fig. 3:**
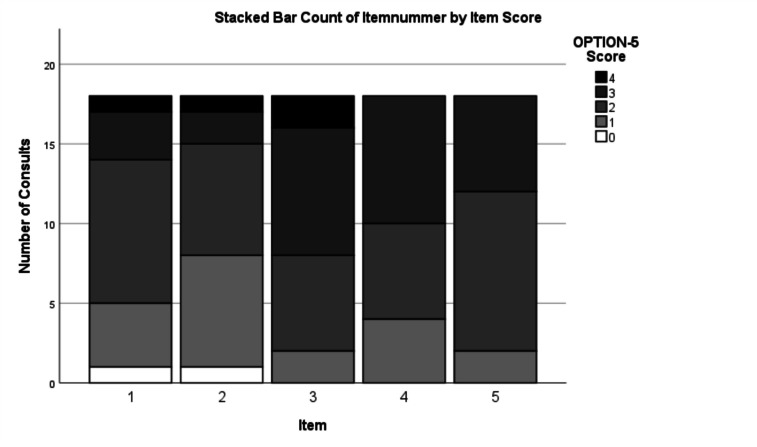
Option-5 item scores: awareness that a decision needs to be made (item 1), assurance that the patient will be well-informed to decide together (item 2), explanation of treatment options including risks and benefits (item 3), elicitation of patients’ preferences (item 4) and deciding together about the most appropriate treatment option (item 5)

**Fig. 4:**
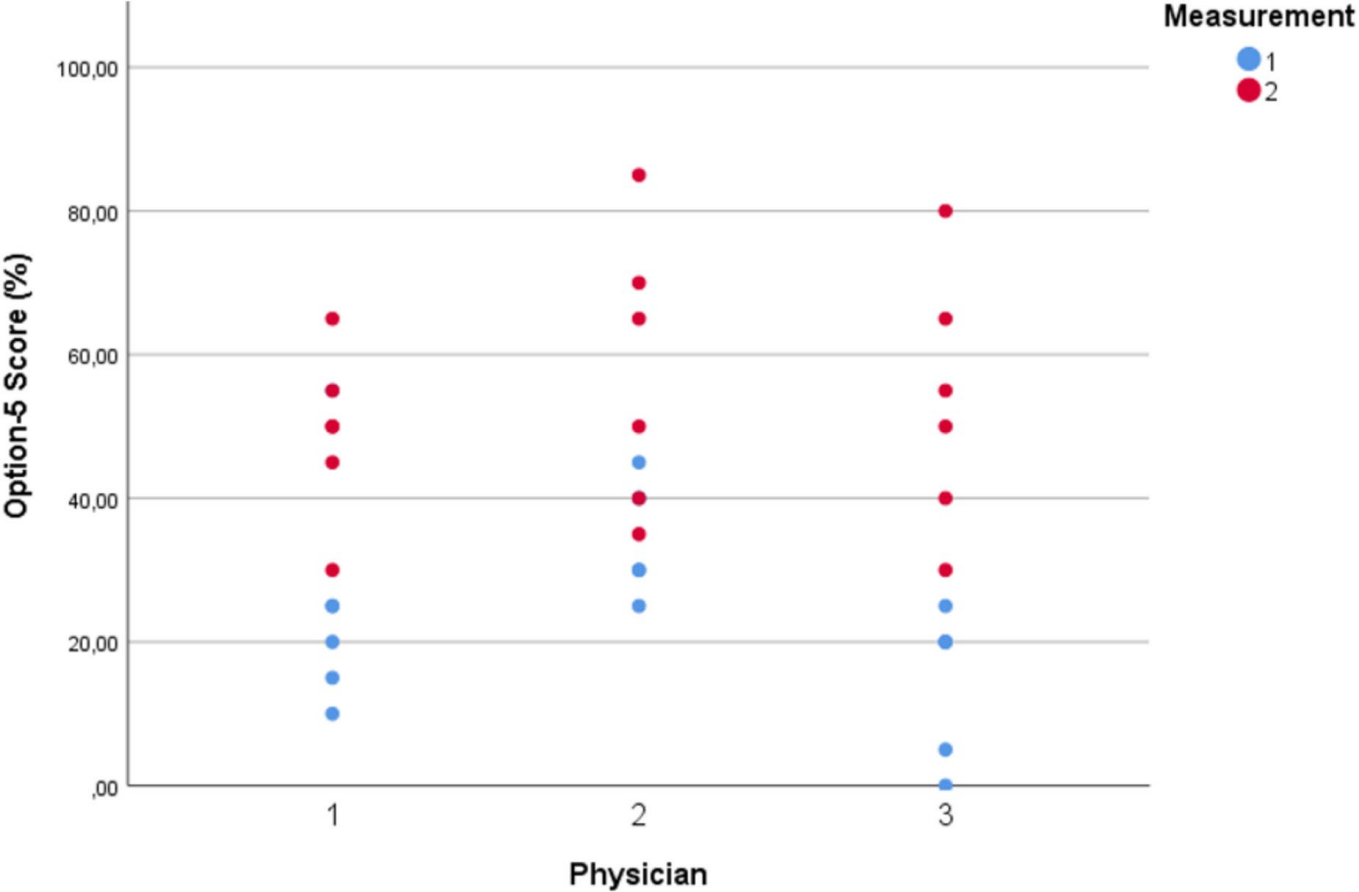
Option-5 boxplot individual scores: 2023 baseline-measurement scores [13] (blue) compared to 2024 post-intervention scores (red)

Interrater reliability yielded a *kappa* value of 0.93 (95%CI 0.83–1.0) after measuring one set of 6 conversations. Median total OPTION-5 score was 50%, IQR 40–65%; range 30–85% (mean score 53.3%, SD 15.9%). Items 3 (‘Informing about the options with their pros and cons’), 4 (‘Exploring patients’ wishes, worries, and expectations’), and 5 (‘Taking patients preferences into account while making the final decision’) were addressed relatively well, as opposed to item 2 (‘Assuring the patient that he/she will be properly informed to make a balanced decision about the preferred treatment option’) (Figs. 3 and 4). The teach-back technique to verify the patients and parents’ understanding of the information as provided by the paediatrician, was rarely observed.

### SDM-Q-9 and SDM-Q-Doc

**Fig. 5:**
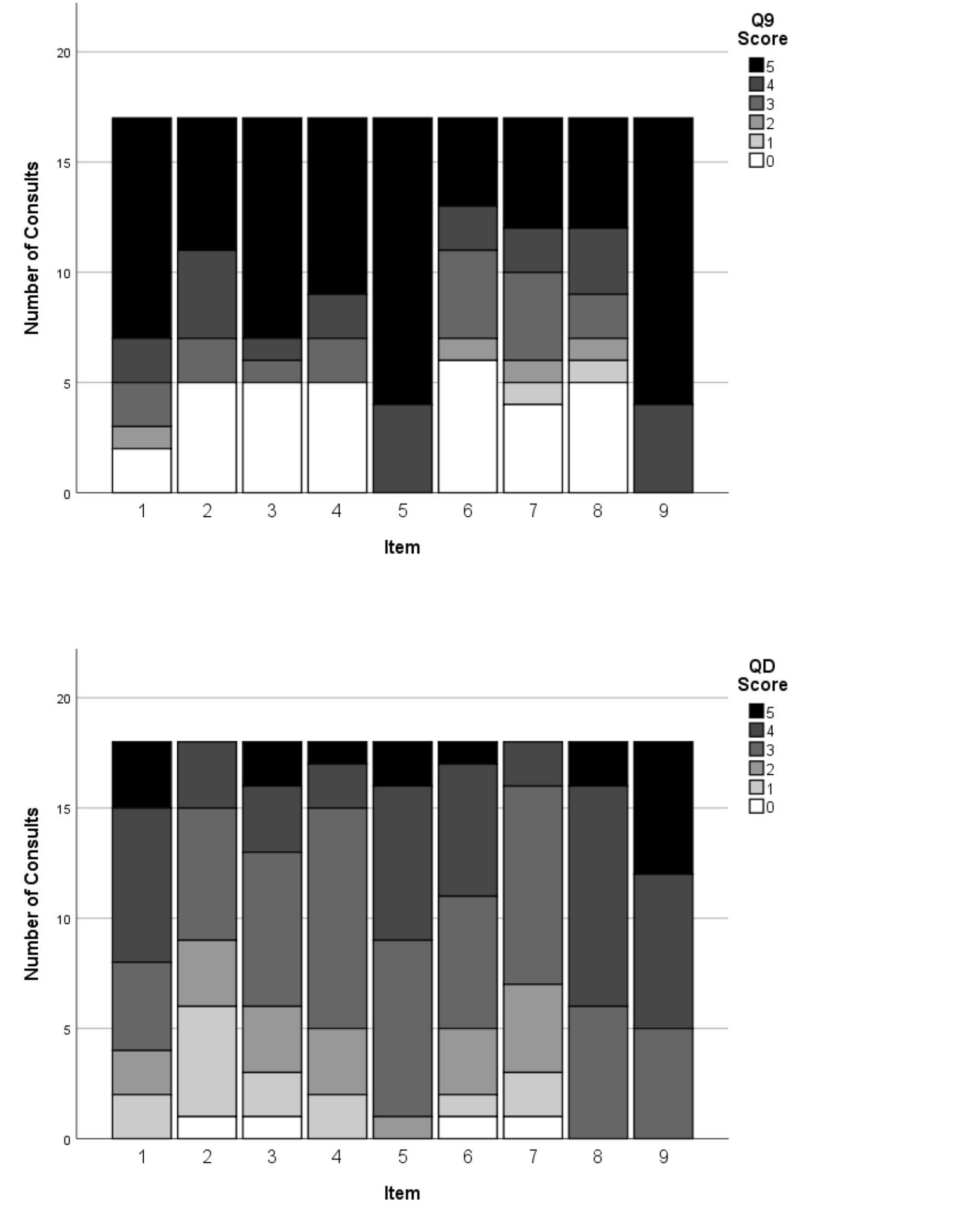
**a** and **b** SDM-Q-9 and SDM-Q-Doc total scores per item, respectively. 1, Clarifying that a decision needs to be made. 2, Eliciting patients involvement preferences. 3, Clarifying multiple ways to deal with the health-related problem. 4, Explaining the pros and cons of each (non)treatment option. 5, Patients’ level of understanding of the provided information. 6, Identifying patients preferred (non)treatment option. 7, Weighing of the discussed (non)treatment options. 8, Choosing a treatment option together. 9, Shared agreement on follow-up arrangements

The missing scores from one incomplete SDM-Q-9 from were left out of the analysis. The patients’ median SDM-Q-9 score was 73.0% (IQR 52.2–91.0), range 22.2–100%, while 2 out of 18 participants (11.9%) gave the maximum score (100%) (see Fig. 5a). Overall, median SDM-Q-9 scores were significantly higher than the SDM-Q-Doc scores (*p* < 0.01). High scoring SDM-Q-9 items were items 5 (‘Investigating if the patient has understood the information’) and 9 (‘Agreement on follow-up arrangements’). Items 6, 7, and 8 (‘Exploring patient/parents’ preferred treatment option’ and whether patient and/or parent were involved by the paediatrician in the process of weighing the pros and cons of different treatment options, and the child/parent’s level of involvement when choosing a treatment option together with the paediatrician, respectively) showed most room for improvement.

The paediatricians’ median SDM-Q-Doc score was 62.2% (IQR 55.6–71.1%, range 44.4–80%) (Fig. 5b). High scoring items were item 1 (clarifying that a health-related decision needed to be made), 8 (Appreciating the paediatricians’ perception of involving the child/caregiver when choosing a treatment option together) and item 9 (‘Agreement on follow-up arrangements’). Items 2 (‘Eliciting the patients preferred involvement in the decision-making’) and 6 (taking the patient/parents’ preferred treatment option into account) scored lowest.

### Bland–Altman analysis

**Fig. 6:**
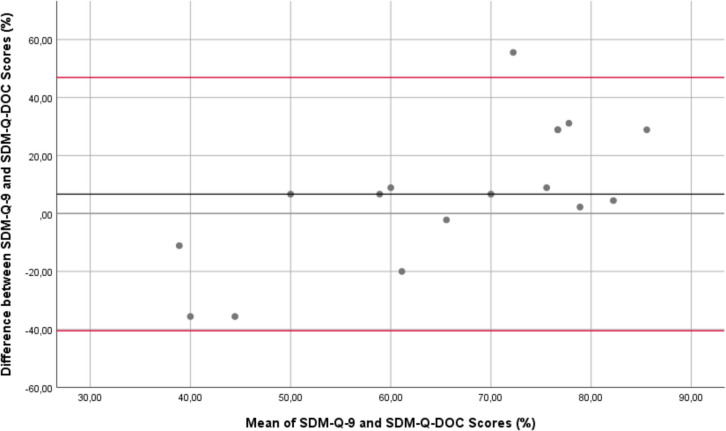
Bland–Altman plot of the differences between SDM-Q-9 and SDM-D-doc against the mean values. The black horizontal line indicates the mean difference between the Q-9 and the Q-Doc scores. The red lines indicate the 95% limits of agreement between Q-9 and the Q-Doc scores

The Bland–Altman plot showed that individual SDM-Q-9 scores were systematically higher than the SDM-Q-Doc scores (mean difference 6.7%, with wide 95% limits of agreement (− 35.6 to + 55.6%). The plot also showed a trend towards a higher difference in SDM-Q scores (Q-9 minus Q-Doc) with increasing mean scores (Fig. 6). This indicates that with increasing scores, patients and caregivers were systematically more confident that they were being involved in the decision-making process by the paediatrician, compared to how the paediatricians themselves perceived their own ability to involve the patient/caregiver in a shared decision-making process.

## Discussion

In this study the level of shared decision-making among paediatric patients with SCD was assessed after groupwise and individual training of the paediatricians, and introducing an SDM tool for the children. This led to a fairly high observed level of SDM, while perceived levels of SDM were high.

When comparing these results with our earlier baseline measurement in a very similar patient population with the same paediatricians [16], the observed level of SDM increased substantially from a median of 25 to 50% on the OPTION-scale. This translates into an improvement from a ‘low’ to a ‘moderate’ effort to engage patients/parents in the decision-making process. These effects are similar to other areas in medicine, like breast cancer [30], and vascular surgery [31, 32]. While the SDM-promoting training and tools have likely contributed to this improvement, a growing awareness amongst the team of paediatric haematologists about the added value of SDM and their willingness to apply SDM in clinical encounters may also have stimulated their effort to involve patients/caregivers in decision-making processes.

Our study design did not allow us to identify which of the interventions led to the improvements in level of SDM, Still, we suggest that active involvement of participants through training and decision support tools is essential to increase the level of SDM during clinical encounters, as studies in other medical realms pointed in the same direction [30, 32, 33].

The ‘3 good questions’ cards address several aspects of SDM, like the paediatricians’ effort to make the patient aware that a health-related decision needs to be made for which there are various treatment options available, deliberation about various treatment options and the risk and benefits of each of these options and can serve as a reminder for the paediatrician to elicit the child and parents’ preferences, worries, and expectations.

Obviously, the level of child involvement is a gliding scale, depending on its age. The higher the child’s age, the less parents may be involved in the decision-making process. Based on the audio-recordings we observed effort from paediatricians to involve children younger than 12. Even though caregivers of these children are legally entitled to be the designated substitute (shared) decision-makers on behalf of the child. Within this context and since our study focus on the level of SDM between paediatric haematologists on the one hand and children and their caregiver on the other, we included all variations of shared decision-making between patient/caregiver on the one hand and paediatricians on the other. Other studies in the Netherlands seem to indicate that involving young children in a decision-making process is not standard procedure [34].

An important observation was that the *teach back* technique, that has proven to be useful to verify the patients and parents’ understanding of the information provided [35], was rarely observed, even though it was thoroughly discussed during both the individual- and team SDM training and patients and caregivers felt well-informed by the paediatrician as the SDM-Q-Doc results showed. We did not, however, assess the patients’ knowledge they had retained about the information they received from the clinician. To understand the goal and added value of SDM, introducing and explaining the ‘3 good questions’ cards may suit the pivotal role the nursing staff play in a paediatric haematology clinic. Their contribution could help further improve the level of SDM [36–40].

Patients and their parents perceived more SDM than the paediatricians did, especially in the higher range of OPTION scores. Apparently, when OPTION scores increased patients and parents perceived more participation in the decision-making process, whereas paediatricians seemed to have become more aware of their limited SDM skills.

Regarding consultation duration, a longer duration might allow for a better shared decision-making process. However we did not find a difference in consultation duration before and after the intervention. Moreover, literature shows that applying SDM does not require longer consultation duration [41].

### Study limitations

The OPTION-5 instrument has not yet been validated for a triadic decision-making process and has been limitedly used in paediatric settings [23, 31, 42, 43]. However, given its strong psychometric properties and the general applicability of SDM as a collaborative decision-making technique, we deem it a valuable method of measuring SDM in a paediatric setting.

Another limitation of our study is the small number of participating paediatric haematologists as well as the single centre setting, which limits the generalizability of the results. However, there are no reasons to suspect that our results are not applicable to other paediatric centers or diseases.

Furthermore, despite the SDM-Q-9 scales’ strong internal consistency [44], it cannot be ruled out that the SDM-Q-9 is sensitive to child and parents’ vulnerability towards authority and/or expert halo bias [42], so that patients score satisfaction with their physician and care rather than the level of perceived SDM. Also, the possibility of paediatricians’ unawareness of their own bias regarding the child’s and parents’ levels of health literacy could have impacted their SDM-Q-DOC scores, since language- and cultural barriers are known factors to impact the physicians’ effort in engaging the child and/or caregiver in the decision-making process [45, 46]. Finally, social desirability bias cannot be ruled out because of the researcher’s presence in the same room with patient and or caregiver when the SDM-Q-9 questionnaire was completed.

### Implications for clinical practice and future research

The results of our post-intervention study are promising. However, due to the small sample size further research is needed, preferably in a larger population, to explore the impact interventions like groupwise and individual training have on the level of SDM. Also, studies are needed to explore the impact of implementing the *3 Good Questions* decision support tool in clinical practice. These effects should be studied both separately as well as in combination in order to determine if, and to what extent, these interventions may strengthen each other.

## Conclusion

SDM training and the use of the ‘*3 Good Questions for Children*’ tool appear to be useful to promote the level of involvement in the decision-making process of children with SCD and their parents. Future studies need to determine to what extent interprofessional training methods for paediatric clinicians and *the* ‘*3 Good Questions*’ for patients will lead to more SDM, which in turn may improve health outcomes.

## Data Availability

All research data available upon request.
